# Charge State Influence on Stability and Isomerism in Dehydrogenated PAHs: Insights from Anthracene, Acridine, and Phenazine

**DOI:** 10.1002/cphc.202400729

**Published:** 2024-12-29

**Authors:** Khaldia Zghida, Farouk Hamza Reguig, Manuel Alcamí, Al Mokhtar Lamsabhi

**Affiliations:** ^1^ LCPM Laboratory Faculty of Exact and Applied Sciences Chemistry Department University of Oran 1 Ahmed BenBella Oran 31000 Algeria; ^2^ Departamento de Química Módulo 13 Universidad Autónoma de Madrid Madrid 28049 Spain; ^3^ Institute for Advanced Research in Chemical Sciences (IAdChem) Universidad Autónoma de Madrid 28049 Madrid Spain

**Keywords:** PAH, Dehydrogenation, Ionization, IR-spectra, Isomerization

## Abstract

In this study, we systematically explored the stability and isomerism of neutral and dehydrogenated polycyclic aromatic hydrocarbons (PAHs) in various charge states, focusing on anthracene, acridine, and phenazine. Our findings highlight key aspects that deepen the understanding of these molecules’ reactivity and stability, relevant in both laboratory and astrophysical contexts. Structural symmetry and the presence of nitrogen atoms significantly impact PAH stability and reactivity. The optimal site for the first dehydrogenation varies with charge state, with notable differences in stability observed across different positions and charge states. For the loss of two hydrogens, there is a clear competition between low and high spin states, influenced by the positions of the hydrogens lost. Infrared spectral analysis reveals characteristic frequencies of conjugated C_sp2_−C_sp2_ bonds and variations across different charge states. The elimination of H_2_ typically occurs at adjacent carbons, forming bonds similar to triple bonds. Reaction networks for anthracene, acridine, and phenazine indicate preferred pathways for hydrogen loss, driven by the need to minimize charge repulsion and maintain aromaticity. Adjacent hydrogen loss is predominant in neutral and singly charged states, shifting to non‐adjacent loss in higher charge states.

## Graphical abstract

The ionization of anthracene, acridine, and phenazine has been explored, along with the removal of one and two hydrogen atoms, to provide valuable information for astrochemists and to understand the behavior of the resulting structures and their electronic reorganization.

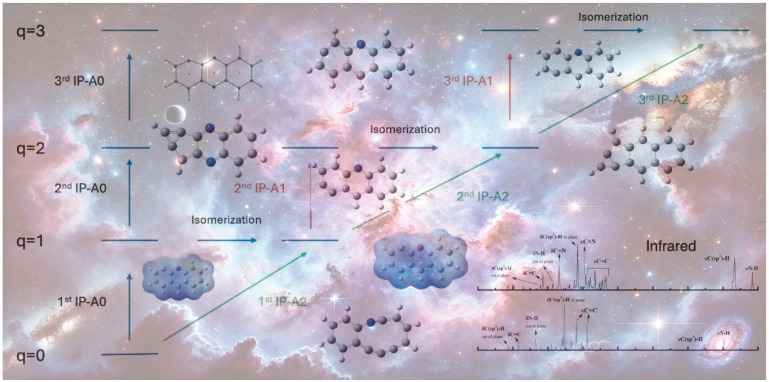

## Introduction

Polycyclic Aromatic Hydrocarbons (PAHs) represent a complex category of organic compounds. Over the past few decades, their significance has grown substantially in various scientific fields, particularly in astrophysics and astrochemistry. They are considered to be a crucial component of interstellar dust and gas.^[1]^ The detection of PAHs in the interstellar medium[^2^, ^3^] (ISM) has piqued the interest of many scientists, leading to extensive research into their origins and chemical reactivity. Moreover, their presence as environmental pollutants add urgency to the need for further research and mitigation.^[4]^ PAHs can originate from both natural and anthropogenic sources, including the incomplete combustion of organic matter from natural phenomena such as forest fires and volcanic eruptions,[^5^, ^6^] as well as human activities like engine emissions, industrial production, waste incineration, and tobacco smoke, among other synthesized materials. Most PAH molecules have a planar structure, comprising multiple fused rings of carbon atoms, with a benzene‐like decoration of hydrogen atoms around their exterior perimeters. They can exist in numerous isomeric forms, many of which are known for their toxic, carcinogenic, and mutagenic properties.[^7^, ^8^]

Polycyclic Aromatic Nitrogen Heterocycles (PANHs), which are nitrogen derivatives of PAHs, have also proven to be significantly relevant in the field of chemistry. Notably, nitrogen is the fourth most abundant chemically reactive element in space and can replace a CH group in the aromatic bonding of the molecule, thereby contributing to the robustness of the PAH structure. Increasing evidence suggests that these PANHs might be present in space.[^1^, ^2^, ^5^, ^9^, ^10^] In fact, both PAHs and their nitrogen derivatives have been detected in meteorites[^11^, ^12^, ^13^] and are believed to be a component of Titan's atmospheric haze.[^14^, ^15^] The inclusion of PAHs in the Interstellar Medium (ISM) inventory could have profound astrobiological implications, given that PAH molecules play a role in various biogenic processes. Therefore, the existence of PANHs in the ISM would suggest their availability to habitable bodies throughout the universe.^[16]^

The fragmentation patterns of various PAH molecules have been explored both experimentally, employing techniques such as photodissociation,[^17^, ^18^, ^19^, ^20^, ^21^, ^22^, ^23^, ^24^, ^25^] collision‐induced dissociation,[^26^, ^27^, ^28^, ^29^, ^30^, ^31^, ^32^] and theoretically, utilizing methods like density functional theory (DFT) calculations,[^33^, ^34^] electron impact ionization,[^35^, ^36^] and fast atomic bombardment.^[37]^ Most of these investigations focused on neutral and singly charged molecules, revealing that the most predominant decay pathways, typically involving the loss of H, H^+^, H_2_, H_2_^+^, or acetylene molecules (C_2_H_2_), are largely independent of the PAH size. Furthermore, these fragments often attach themselves to larger molecules, illustrated in cases involving biomolecular ions in water nanodroplets,,[^38^, ^39^] molecules and clusters in helium nanodroplets,[^40^, ^41^] clusters of fullerenes, PAHs and/or biomolecules, and electrospray ionization.[^27^, ^42^, ^43^, ^44^, ^45^, ^46^, ^47^, ^48^] The latter process is particularly noteworthy, as solvent molecules provide a protective layer, preventing large and fragile biomolecules from fragmentation.^[49]^ This phenomenon can be attributed to the rapid redistribution of charge and excitation energy across a larger system, endowed with additional internal degrees of freedom. This redistribution has been empirically demonstrated even in instances where the initial interactions were strongly localized.[^17^, ^50^] It's within this context that the hydrogenation of PAHs and PANHs occurs.

To ascertain the IP (IP) of PAH molecules, an array of experimental methods has been utilized.^[51]^ In a notable study by Denifl and colleagues in 2006, the first, second, and third IPs of PAHs were measured through an electron ionization process with an energy of 100 eV, using a high‐resolution detection method.^[40]^ The most recent data on the first and second IPs of PAHs can be found in the NIST Chemistry WebBook.^[52]^ Nonetheless, it's challenging to find results for trications or tetracations, especially for larger PAH molecules such as Coronene. Vibrationally excited molecules can undergo two competing processes: IR emission and fragmentation.^[53]^ These processes operate on different time scales. Fragmentation occurs on a timescale of a few picoseconds (*ps*) and typically results in the loss of H, H_2_, or C_2n_H_x_, dominating the experimental mass spectra of PAH fragmentation.[^54^, ^55^, ^56^] These patterns also emerge in recent studies on ion‐induced fragmentation and molecular.[^25^, ^43^, ^57^, ^58^, ^59^]

**Figure 1:**
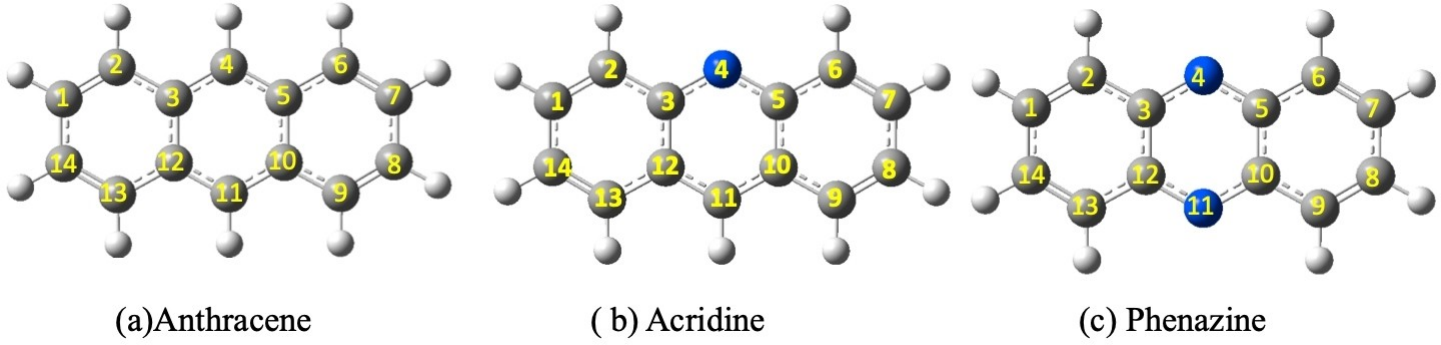
structure of anthracene C_14_H_10_, acridine C_13_H_9_N and phenazine C_12_H_8_N_2_. With the numeration of atoms used in this article.

A theoretical model aimed at determining the ionization energies of aromatic hydrocarbons has been suggested.^[46]^ This model, particularly pertinent to large PAHs, views the ionization of PAH molecules as akin to ionizing fragments of graphite. Consequently, their IPs converge towards the sum of the graphite work function (V_gr_) and the electrostatic work required to charge a conductor shaped like the molecule. This concept was further utilized recently to ascertain the first and second theoretical IPs for several PAHs, including naphthalene, biphenylene, anthracene, pyrene, and coronene.^[24]^ Notwithstanding these experimental and theoretical strides, there remains an information gap concerning the IPs (IP) of PAHs. In this study, we aim to fill this void by presenting a theoretical analysis of the 1^st^, 2^nd^, and 3^rd^ IPs for anthracene (C_14_H_10_), acridine (C_13_H_9_N), and phenazine (C_12_H_8_N_2_), as well as their dehydrogenated isomers (see Figure 1). Moreover, we will investigate their corresponding molecular cations that result from the elimination of one or more electrons, and how this impacts their thermodynamic stability.

Understanding the stability of chemical compounds in laboratory chemistry is crucial for interpreting reaction pathways and the structures of the involved reactants. In our study, we aim to identify unstable structures that emerge when we dehydrogenate PAHs, with an emphasis on their relevance in astrophysics and outer space. It is theorized that the high‐energy collisions occurring in these regions could yield structures that are inconceivable in a laboratory setting, and these could potentially contribute to the creation of compounds detectable by astrochemists. An intriguing line of inquiry pertains to the spin states of these structures when we remove 1, 2, or 3 electrons, and also when we eliminate 1 or 2 hydrogens. This information, though currently scant in scientific literature, could hold substantial value for astrochemists. By delving into these less explored questions, we hope to further illuminate the intricate processes at play in the vastness of space, expanding the frontiers of our knowledge in astrochemistry.

## Computational Details

Our results are based on density functional theory calculations as implemented in the GAUSSIAN 16 package.^[60]^ We employed the B3LYP functional which combines Becke's three‐parameter nonlocal hybrid exchange potential with the non‐local gradient corrected correlation function of Lee, Yang, and Parr.[^61^, ^62^] The computational strategy adopted aligns with the methodologies of recent studies on similar compounds, which facilitates the sharing of insights and the derivation of coherent conclusions.[^33^, ^63^, ^64^, ^65^, ^66^, ^67^, ^68^, ^69^, ^70^, ^71^] It has been established that the B3LYP method can accurately describe both the infrared^[63]^ and UV‐visible spectra^[72]^ of PAHs, as well as their interactions with other compounds, such as methane.^[73]^ The IPs, which are also part of our study, have been compared with experimental values, at least for the first ionization. The trends observed are consistent between our calculations and the experimental data, as will be further detailed in the manuscript.

Geometric optimization of neutral and charged structures was performed in different spin multiplicities using the 6–31G (d,p) basis set. Frequency calculations have been carried out to ensure that local minima are obtained. Single point energy calculations were also performed using larger 6–311++G (3df, 3pd) basis set. Zero‐point energy correction (ZPE) has also been included in the energy of optimal geometries for the calculation of adiabatic IPs, relative stabilities and dissociation energies.

Same level of calculations was used for the neutral, singly‐, doubly‐ and triply charged anthracene, acridine and phenazine structures and 2 spin multiplicities have been taken into account corresponding to the lowest spin states (singlets a triplet for neutral and doubly charged species, and doublet and quadruplet for singly and triply charged species).

For the ionization energy hybrid functionals can lead to relevant errors, therefore we refined the energies obtained by conducting a single‐point calculation with the coupled cluster method considering singlet, doublet, and triplet states. Given the computational cost, we used the domain‐based pair natural orbital coupled‐cluster approximation (DLPNO‐CCSD(T)) to enhance calculation efficiency.[^74^, ^75^] This method includes electron pairs with significant contributions in the calculation, while treating remaining pairs at the MP2 level or disregarding them.[^76^, ^77^, ^78^] For our systems, this approach yields results closely aligned with experimental values at a reduced computational expense. Calculations utilized the triple‐zeta correlation‐consistent basis set, cc‐pVTZ. Additionally, we extrapolated the coupled cluster energies to the Complete Basis Set (CBS) limit using three‐point extrapolation based on cc‐pVDZ, cc‐pVTZ, and cc‐pVQZ with corresponding auxiliary basis sets, as implemented in ORCA (version 6.0).^[79]^

## Results and Discussion

### Relative Stability

As mentioned above, the main objective of this study is to elucidate the behavior of anthracene, acridine, and phenazine when they are singly, doubly or triply ionized to three electrons and lose of 1 or 2 hydrogens. This requires investigating these systems with total charges 0–3, both in their non‐dehydrogenated molecule and after losing one or two H. The various compounds under study exhibit several possibilities for the loss of the first H, but many of them are equivalent due to symmetry. Anthracene, having 14 H susceptible to dissociation, only three positions need to be considered: 1, 2, and 4 (see Figure 1). In phenazine, with no H atoms at positions 4 and 11, only the losses at positions 1 and 2 needs consideration. For acridine, as it presents a lower symmetry, the H dissociation at positions 1, 2, 8, 9, and 11 must be considered. For the loss of the second H the situation is more complex as the first loss implies a loss of symmetry, in the three molecules all the non‐equivalent possibilities have been systematically studied. Their relatives energies, evaluated at the B3LYP/6‐311++G(3df,2p)//B3LYP/6‐31G(d,p) level of theory, with respect the most stable form for each charge and number of H lost are summarized in Tables 1 (for anthracene), 2 (acridine) and 3 (phenazine). In these tables the positions of H loss are numbered following the notation of Figure 1. A data set collection of computational results is available in the ioChem‐BD repository^[80]^ and can be accessed via https://doi.org/10.19061/iochem‐bd‐6‐387.

**Table 1:**
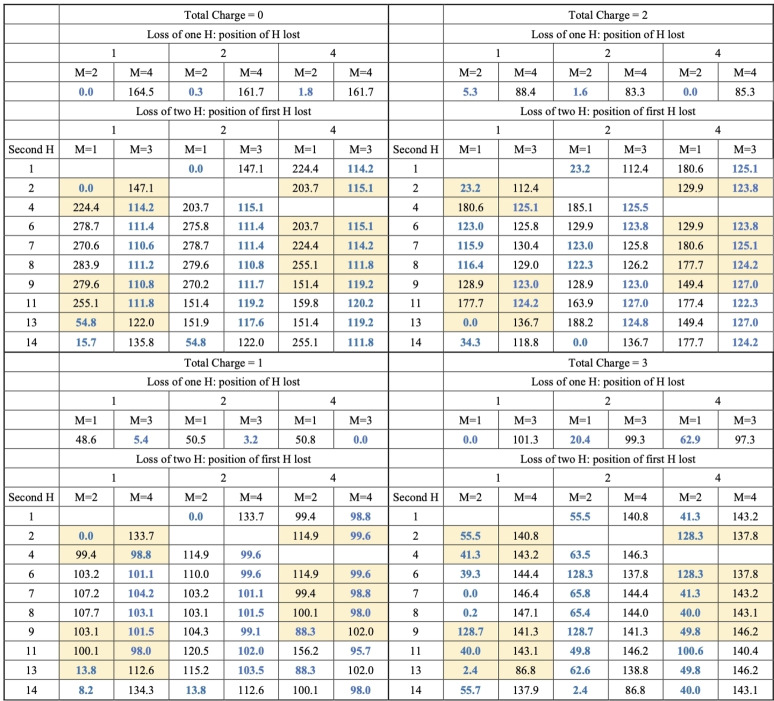
Relative energies (in kJ/mol) for all the structures obtained for Anthracene after losing 1 or 2 hydrogens. Relative energies refer to the most stable isomer with the same number of H and same charge. M refers to the Spin Multiplicity. The most stable values of multiplicity are highlighted in dark blue. Yellow background indicates that this form is equal by symmetry to other in white background.

**Table 2:**
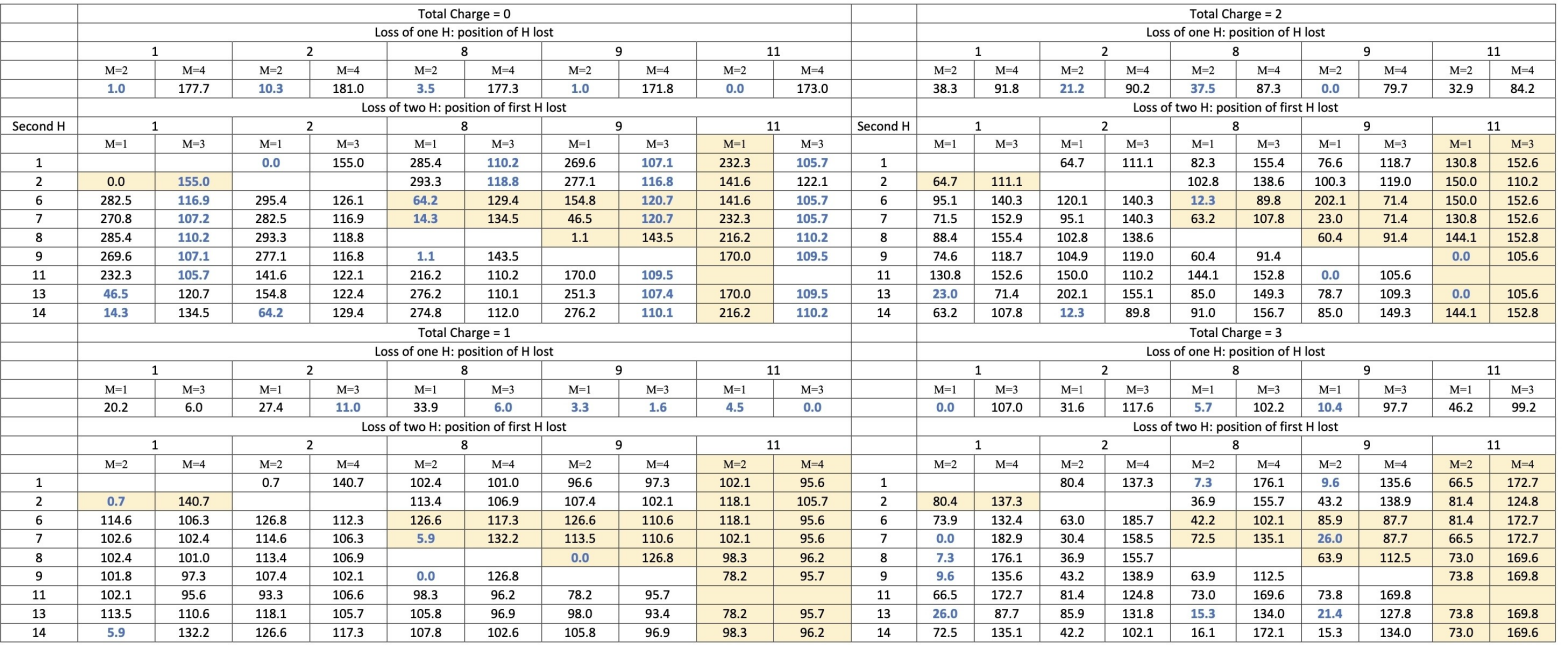
Relative energies (in kJ/mol) for all the structures obtained for Acridine after losing 1 or 2 hydrogens. Relative energies refer to the most stable isomer with the same number of H and same charge. M refers to the Spin Multiplicity. The most stable values of multiplicity highlighted in dark blue. Yellow background indicates that this form is equal by symmetry to other in white background.

**Table 3:**
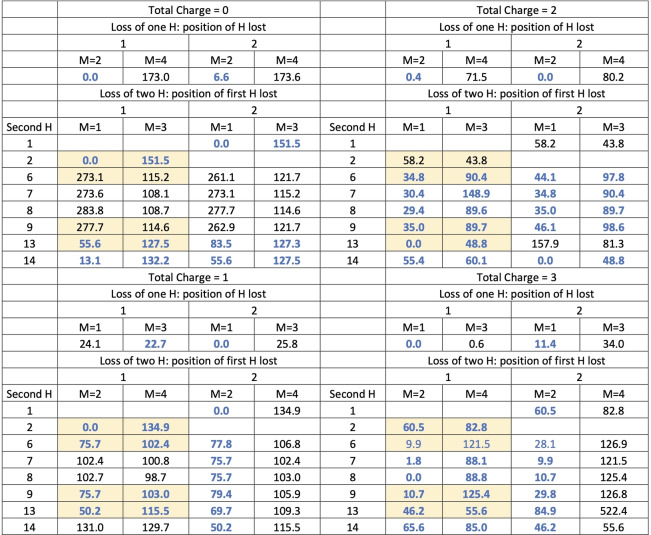
Relative energies (in kJ/mol) for all the structures obtained for phenazine after losing 1 or 2 hydrogens. Relative energies refer to the most stable isomer with the same number of H and same charge. M refers to the Spin Multiplicity. The most stable values of multiplicity are highlighted in dark blue. Yellow background indicates that this form is equal by symmetry to other in white background.

The first observation is the critical importance of high multiplicity when considering all possible configurations for dehydrogenation. For the loss of a single hydrogen atom with q=0, 2 and 3 it's consistently found that the most stable configuration is the one having the lowest multiplicity: a doublet for q=0, 2 and a triplet for q=3.

**Figure 2:**
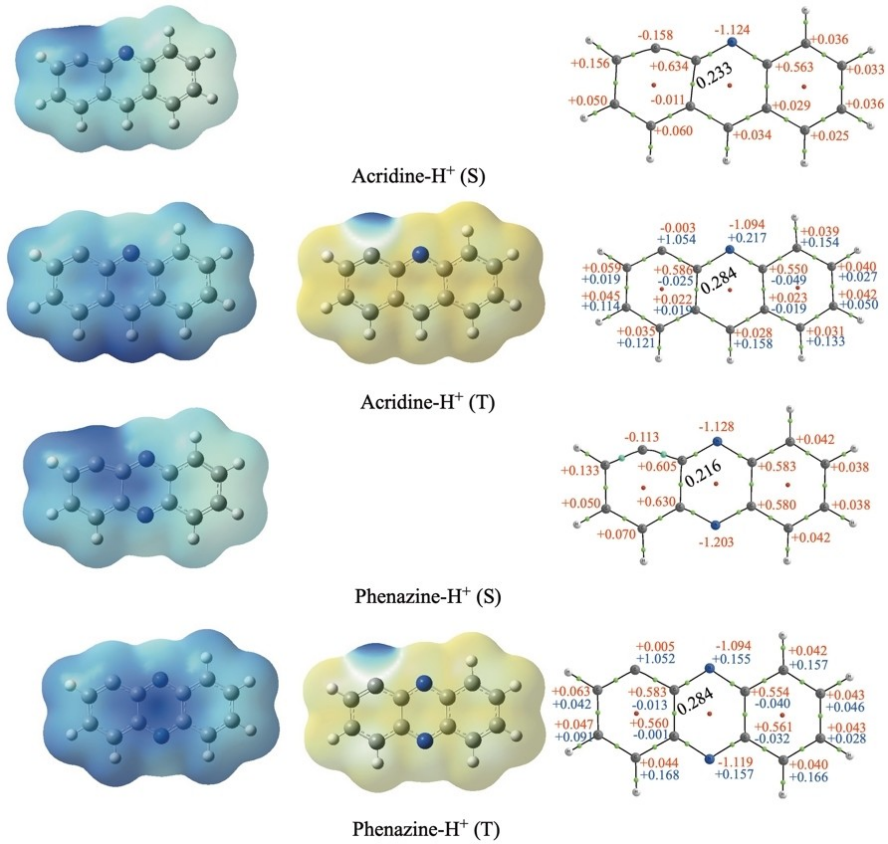
Electrostatic potential graph (left), spin density graph (center), and QTAIM molecular graph (right) for positively charged dehydrogenated acridine and phenazine. First numbers, in red, corresponds to the charges, for triplets second values corresponds to spin values highlighted in blue, and BCP electron density for the C3−C12 bond presented in black, all expressed in a.u.

On the contrary, for q=1, triplet states dominate in terms of stability, attributable to one unpaired electron arising from the breaking of the C−H bond and the other from ionization and pointing out that the location of the charge does not coincide with the point of hydrogen loss. We have analyzed in more detail the case of acridine and phenazine when hydrogen is removed from position 2, in both the triplet and singlet states (see Figure 2). The carbon affected by dehydrogenation maintains a neutral charge (close to zero) in the triplet state of both compounds and becomes slightly negative in the singlet (around −0.11 e). Regarding spin density in the case of the triplet, in both compounds the distribution across the charge density demonstrates that one spin is concentrated on the dehydrogenated carbon, while the other is distributed among the opposing carbon atoms and the nitrogen atom(s). Singly charged phenazine dehydrogenated at position 2 is the only exception; in this particular case, the singlet becomes the most stable form. The clearest change in the structure upon the lost of one hydrogen is the lengthening of the C3−C12 bond which in turn weakens the resonance in the benzene rings of both molecules, a key aspect for their stability. In anthracene losing one H elongates this bond from 1.444–1.518 (singlet) and 1.453 (triplet). In phenazine it changes from 1.445–1.552 (singlet) and 1.456 (triplet) in this molecule and for symmetry reasons also affect the C5−C10 bond that elongates to 1.457 (singlet) and 1.447 (triplet). Figure 2 shows that these changes are also reflected in the charge density at the C3−C12 bcp. So, the overall effect is that in singly‐charged phenazine in its singlet state, the presence of a second N allows to better accommodate the changes induced by the loss of one hydrogen in position 2 stabilizing it. This is also reflected in Figure 2 in a larger redistribution of the charge along the system in phenazine (singlet) with respect acridine (singlet). One conclusion is that in these systems, with large electronic resonance effects, the analysis of the stability needs to consider the changes in aromaticity, which are not simple to describe and predict.

Regarding the optimal site for the first dehydrogenation, there's a variety of scenarios as we delve into determining the most stable structure for each charge state. For the loss of a single hydrogen with q=0, a near degeneracy is observed in anthracene across all positions. In acridine, all positions are almost degenerate except for position 2, due to the proximity of the nitrogen atom, which strips charge from C2, reinforcing the CH bond. This effect also explains why in phenazine, the loss of hydrogen from position 1 is more favorable than from position 2. When q=1, the loss of hydrogen from the central ring is most stable (position 4 in anthracene and 11 in acridine), this position is favored because of the resonances in the external rings of monocharged species. For phenazine, the loss from position 2 becomes more favorable, as discussed above. For q=2 more pronounced electronic reorganization is observed, anthracene and phenazine maintain the same positions (4 and 2 respectively) as the most stable ones, but in acridine it shifts to 9. For q=3 position 1 is always preferred for single hydrogen loss.

In the case of the loss of two hydrogens, there is a clear competition between low and high spin states depending in first instances from the position from which both hydrogens are lost. As a general rule, for charges q=0 and q=1 when both hydrogens are extracted from the same ring and from positions that differs in one or two C−C bonds, the low spins state are the most stable ones, in all other cases the high spin state is more stable reflecting that electrons are disconnected from each other. For q=2 there is a competition and in general the two states are quite close in energy making more difficult to get general rules to predict the most stable hydrogen loss. This is also the case in acridine and phenazine, the presence of nitrogen atoms produces that now loss and high spin state are more degenerate, and the above rule get more exceptions. For q=3 the low spin state is always the most stable one independently of the positions.

For the removal of two hydrogens, three competing factors seem to influence the outcome: losing hydrogens from the same ring, preserving two aromatic rings, for q=2 and q=3, distributing the charges apart to minimize electronic repulsion and the creation of a new bond when hydrogens are removed from adjacent atoms.

Adhering to these principles, for q=0 and q=1, hydrogens are preferentially lost from positions 1 and 2 (or 8 and 9 in acridine, which are analogous). This results in only one ring losing its aromatic character and the formation of a new bond with partial triple bond characteristics. For q=2, loss from positions 1 and 13 in anthracene and phenazine maintains the aromaticity of two rings while achieving better charge separation; in acridine, positions 9 and 11 are favored. When it comes to q=3, the effect of charge separation takes over, leading to hydrogens being lost from opposite positions 1 and 7 in both anthracene and acridine, and 1 and 8 in phenazine.

### Infrared Spectra

**Figure 3:**
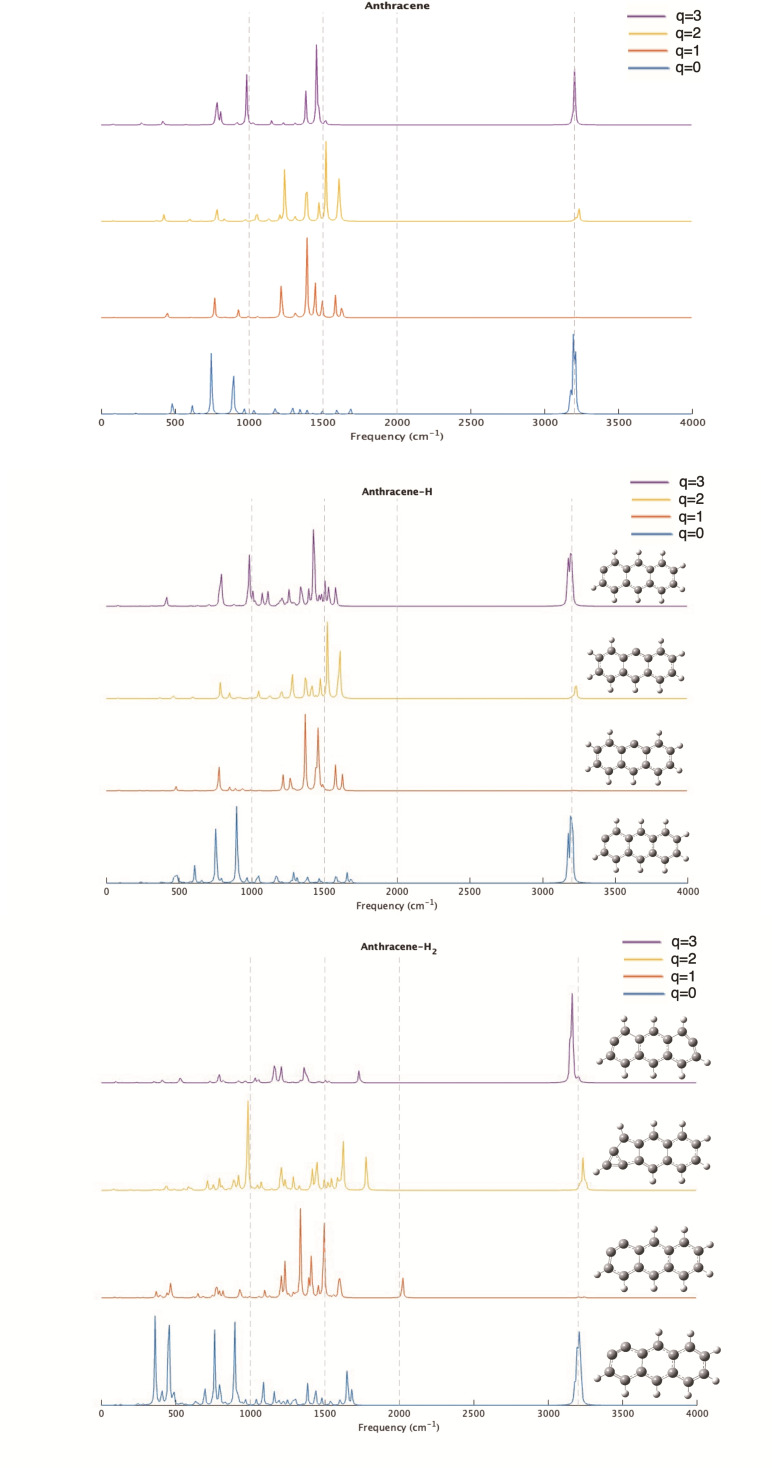
IR spectra of Anthracene and its mono and dehydrogenated homologues at different ionized states (q=0, 1, 2, 3).

**Figure 4:**
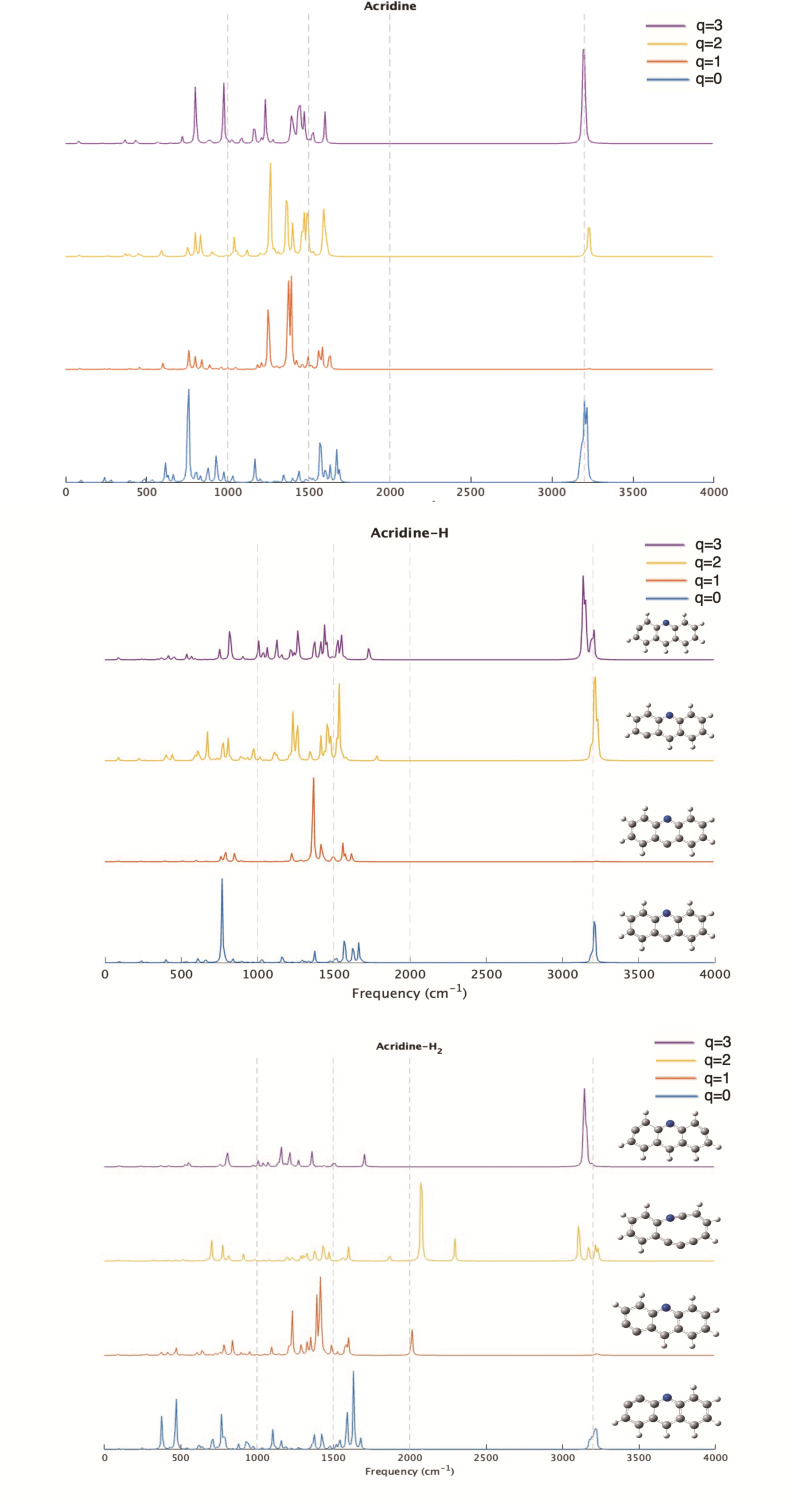
IR spectra of Acridine and its mono and dehydrogenated homologues at different ionized states (q=0, 1, 2, 3).

**Figure 5:**
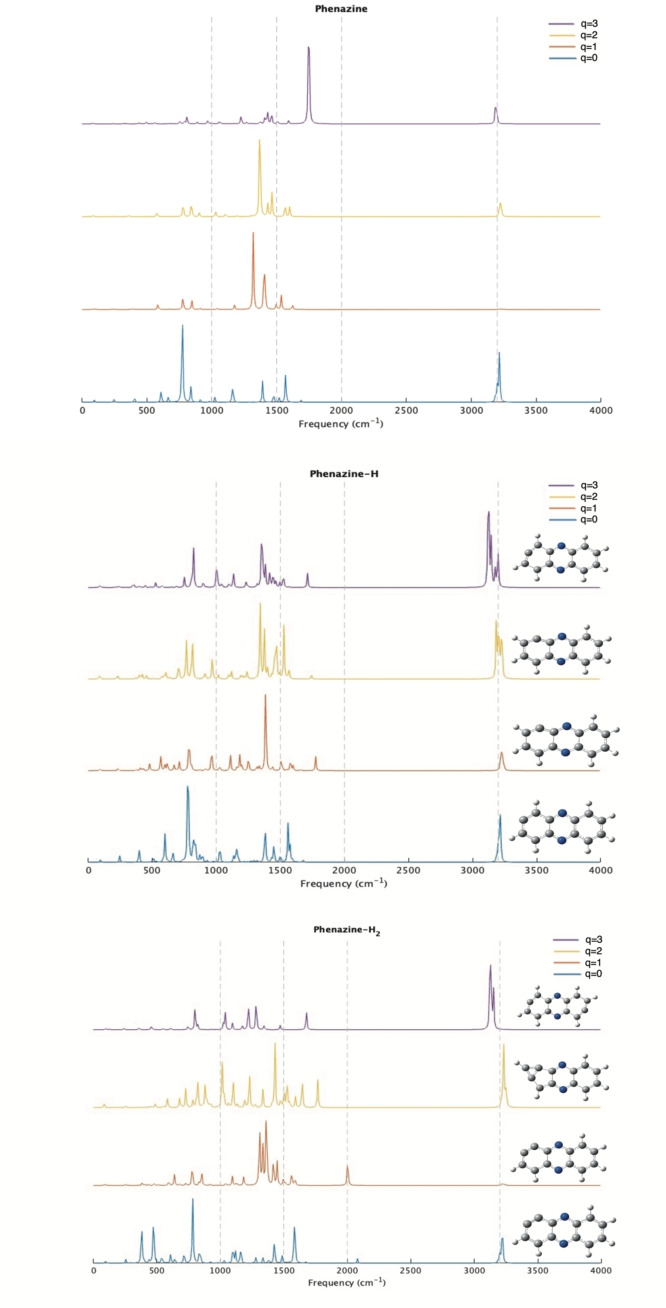
IR spectra of Phenazine and its mono and dehydrogenated homologues at different ionized states (q=0, 1, 2, 3).

One of the most widely used tools for identifying compounds in space is infrared spectroscopy. Having a clear identification enables astrochemists to detect molecules in various distant regions. Anthracene, acridine, and phenazine may share similar spectral characteristics, but they can also exhibit notable variations, as shown in Figures 3, 4, and 5. In this section, we will only discuss the results for the most stable structures and spin multiplicities likely to be observed in distant space. The spectra of all species discussed in the previous section can be downloaded from the IochemBD Database (https://doi.org/10.19061/iochem‐bd‐6‐387.). Ionizing a molecule leads to structural reorganization, which is reflected in changes in bond lengths, thereby influencing vibration frequencies and consequently the vibrational spectrum. Anthracene, acridine, and phenazine are also aromatic molecules, hence there is electronic delocalization across the three rings that compose them. This electronic delocalization is preserved even during ionization, though a significant structural change among the different structures is observed.

The ionization of anthracene, acridine, and phenazine does not result in large changes in the position of their vibrational frequencies, but produces large changes in intensity. The majority of the frequencies associated with C−H, C−C, and C−N vibrations are still observed in the three compounds with slight shifts due to reduced aromatic ring resonance and a decrease in bond strength, thus leading to bond elongation. This is important for astrochemical studies where the identification of potential carriers of unidentified infrared (UIR) emission bands observed in the range of 3–14 micron is a hot topic. When analyzing the 3 μm region (3300 cm^−1^ in the figure), for the neutrals no significant changes are observed with dehydrogenation. The same happens in this region when ionizing the system. This is consistent with the fact that no new CH are formed when dehydrogenating. This is at variance when hydrogenation occurs, in previous studies we observed appearance of new bands in the 3 micron region when coronene is hydrogenated.^[81]^ At 3 μm (2000 cm^−1^) some significant lines appear in anthracene‐H_2_ and acridine‐H_2_ singly charged and specially in acridine‐H_2_ doubly charged, but this is due to the huge change in the ring structure in this latter case. In other regions main changes are due to ionization more than loose of hydrogens.

Therefore, the removal of a hydrogen atom does reveal new vibrational frequencies depending on the molecule and the site from which the hydrogen was removed. It's important to note that our objective is not to address all existing vibration frequencies, but rather to focus on those that exhibit significant differences in their bonds and, consequently, in their frequencies. To conduct this analysis, we have examined the theoretical spectra directly from the output results, as these are not normalized and allow us to distinguish each vibrational mode of the different molecules, even though the intensities may not always be clearly visible in the figures.

In anthracene, two notable structures emerge: the loss of hydrogen from position 1 in the neutral and triply charged states, and from position 4 in the singly and doubly charged states. In both scenarios, frequencies characteristic of conjugated C_sp2_−C_sp2_ bonds are evident, particularly a vibration at 1623 cm^−1^ in the neutral and triply charged states involving exclusively C1 and C2. Additionally, a vibration at 1575 cm^−1^ involving C1/C14 and C7/C8 appears in the singly charged state. The spectral changes for q=1 and q=2 are minimal, indicating that the loss of hydrogen from the central ring (position 4) does not significantly impact the spectral features dominated by the two external rings.

In acridine, the loss of a hydrogen atom leads to three different structures, depending on the overall charge of the molecule. For q=0 and q=1, the most stable structure results from the loss at C11, showcasing a characteristic vibration frequency of 1627 cm^−1^, exclusively associated with the C11 and adjacent C12/10 bonds in the neutral species. Additional vibrations occur at 1570 cm^−1^ and 1560 cm^−1^ for q=0 and q=1, respectively, involving C1/C14 and C7/C8. The vibrational frequency for C1−C2, akin to that observed in anthracene, is noted at 1664 cm^−1^ for q=0 and 1618 cm^−1^ for q=1.

As we move to the dication and trication states, the loss of hydrogen is observed at position 9 for q=2 and at position 1 for q=3. In both cases, a carbon atom with *sp* hybridization appears at different positions, which is reflected in the emergence of a new vibrational frequency in each case. In the dication state, this frequency appears around 1782 cm^−1^ with relatively low intensity, whereas in the trication state, it is blue shifted with a relatively high intensity, appearing near 1731 cm^−1^. (see Figure 4).

The infrared spectral profiles of phenazine missing a hydrogen atom closely resemble those of anthracene‐H when the system's total charge is neutral. The spectra align across almost all frequencies, with minor deviations attributable to nitrogen atoms in phenazine, which are absent in anthracene. For example, the frequency for the C1−C2 bond in phenazine‐H is 1628 cm^−1^, subtly differing by only 4 cm^−1^ from that in anthracene‐H. When phenazine‐H carries a single positive charge and lacks a hydrogen at position 2, its vibrational spectrum distinctly diverges from that of similarly charged counterparts. A significant vibrational frequency emerges at 1776 cm^−1^, involving carbons at the 1 and 3 positions, a frequency also observed in the dication state, albeit blue‐shifted to 1742 cm^−1^. This pattern of vibration, associated with hydrogen loss from the same site, is consistent across different compounds. For instance, the same vibrational shift is noted in the dication state of acridine‐H. In the case of trication phenazine‐H, this scenario mirrors that in acridine‐H, with a characteristic vibration occurring around 1712 cm^−1^ following hydrogen loss at C1. A broader analysis of hydrogen loss in these three compounds across various charge states reveals a consistent pattern: loss of hydrogen at a carbon atom in the outer rings, when charged, disrupts electronic resonance within the molecule. This disruption manifests as a new vibrational frequency range of 1710–1780 cm^−1^, which primarily involves the affected carbon atoms, effectively isolating them similar to an alkene group.

In the case of the removal of two hydrogen atoms, we observe a variety of structures across different molecules. For anthracene, acridine, and phenazine with zero and one charge, the removal of H_2_ occurs at two adjacent carbons, creating a C−C bond that resembles a triple bond. A linear ‐C−C‐ triple bond typically vibrates at 2150 cm^−1^. However, in our cases, the ‐C−C‐ bond is not linear due to its distortion from being part of the ring and is weakened by the loss of an electron in the case of the cation. The vibration frequency of this bond in anthracene‐H_2_ (q=0), acridine‐H_2_ (q=0), and phenazine (q=0) are obtained at 2084 cm^−1^, 2083 cm^−1^, and 2080 cm^−1^, respectively. For molecules with a positive charge, the vibration frequency of this bond shifts to 2021 cm^−1^ for anthracene, 2014 cm^−1^ for acridine, and 2003 cm^−1^ for phenazine. For the dication, the most stable structures result from the loss of two hydrogens at alternate positions, which prevents the formation of a triple bond as in the previous cases but significantly weakens the aromatic ring, leading to the formation of distinct structures. In anthracene and phenazine, dehydrogenation at carbon atoms C1 and C13 leads to the formation of a cyclopropane‐like structure within the molecule in their dication forms. This structural change manifests as a distinct vibrational frequency in the infrared spectrum, observed at 1779 cm^−1^ for anthracene and 1767 cm^−1^ for phenazine. Conversely, in acridine, hydrogen loss alternately affects carbon atoms C11 and C9, resulting in the disruption of the C5 and C10 bond. This alteration engenders the formation of a 10‐membered ring that includes two bond types resembling triple bonds: a carbon−carbon bond and a nitrogen−carbon bond. The respective vibrational frequencies of these bonds in the infrared spectrum are prominently displayed at 2076 cm^−1^, akin to a typical triple carbon bond, and at 2295 cm^−1^, which closely resembles a nitrile bond.

Regarding the triply charged structures, the loss of two hydrogen atoms occurs at symmetrical positions within the molecule. In anthracene and acridine, the hydrogens are removed from positions 1 and 7, while in phenazine, the loss occurs at positions 1 and 8. This pattern results in the three molecules, in their tricationic form without two hydrogen, resembling their trication‐H forms, with the only difference being the removal of a second hydrogen from a symmetrically opposite position. Consequently, the vibrational spectrum of the trications‐H_2_ of the three compounds under study shows similarities to their trication‐H counterparts. As previously mentioned, a vibrational frequency in the range of 1700–1780 cm^−1^ is observed, involving the dehydrogenated carbon atoms.

### Ionization Potential

The adiabatic first, second, and third IPs (IP) for anthracene, acridine, phenazine, and their dehydrogenated isomers have been calculated using the following formula:

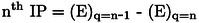




Where E is the total electronic energy at the corresponding level of theory. The subscript q corresponds to the charge.

**Figure 6:**
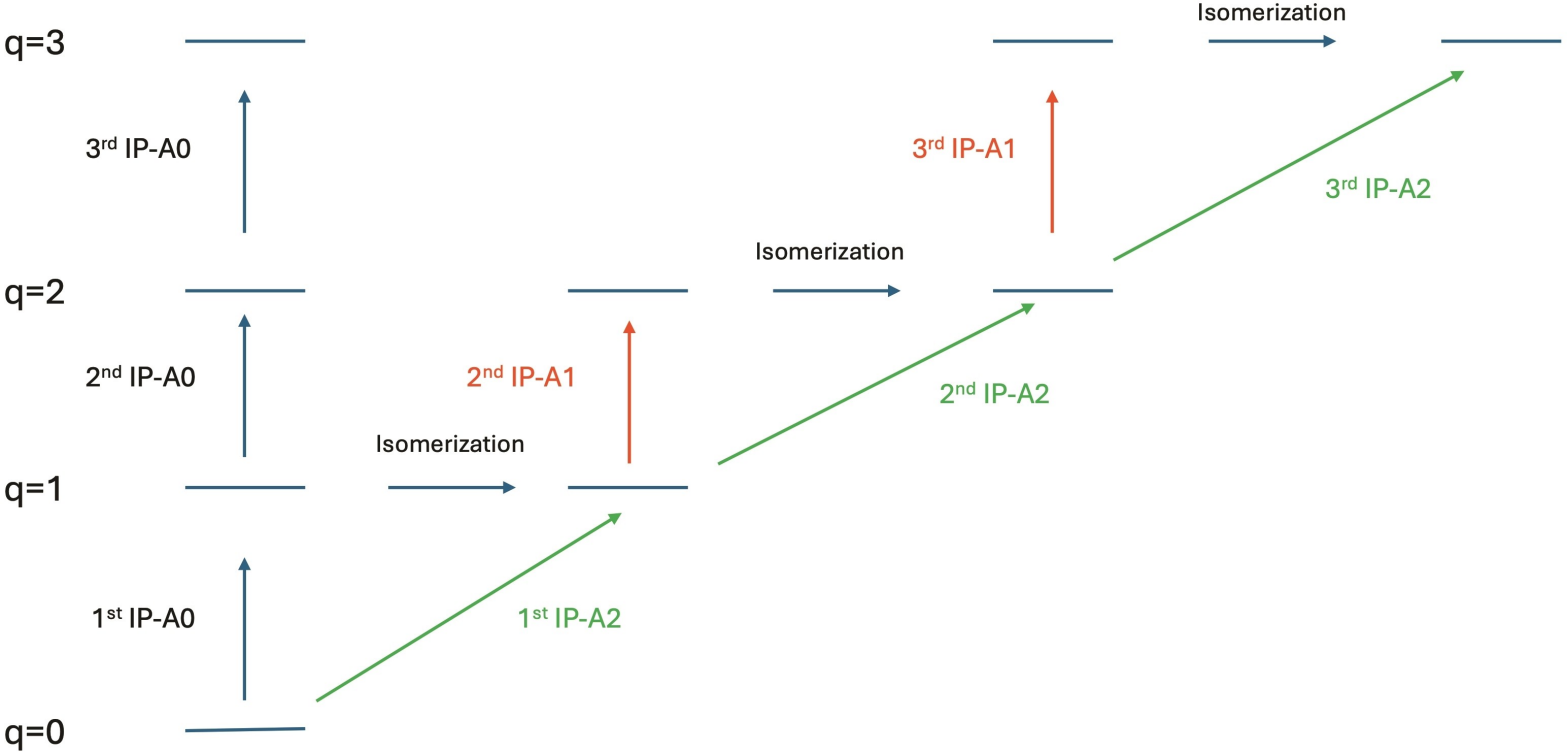
Different definition of Ionization Potentials (A0, A1 and A2) used in this article. Horizontal arrows indicate isomerization, vertical and diagonal arrows indicate ionization.

To analyze the effects separately, we have calculated the IPs in three different ways (see Figure 6):

– We define adiabatic‐0 (IP−A0) as the IP obtained when all hydrogens remain attached to the same carbons as in the most stable dehydrogenated neutral form. This assumes that after ionization, the molecule does not have time to isomerize to the most stable ionized isomer. With this definition, mainly electronic effects are accounted for. Note that these are not vertical IPs, as the structure corresponds to the one optimized in the ionized state.
– Adiabatic‐1 (IP−A1) is defined as the IP obtained when all hydrogens remain attached to the same carbons as in the most stable dehydrogenated q‐1 form. This assumes sequential ionization, allowing the molecule time to isomerize to the most stable structure only after each ionization. This accounts for both electronic effects and structural effects due to isomerization of the lower state.
– Adiabatic‐2 (IP−A2) is defined as the IP obtained when the most stable isomeric form for all the involved species is considered. This accounts for electronic effects and structural effects due to isomerization in both states.

**Figure 7:**
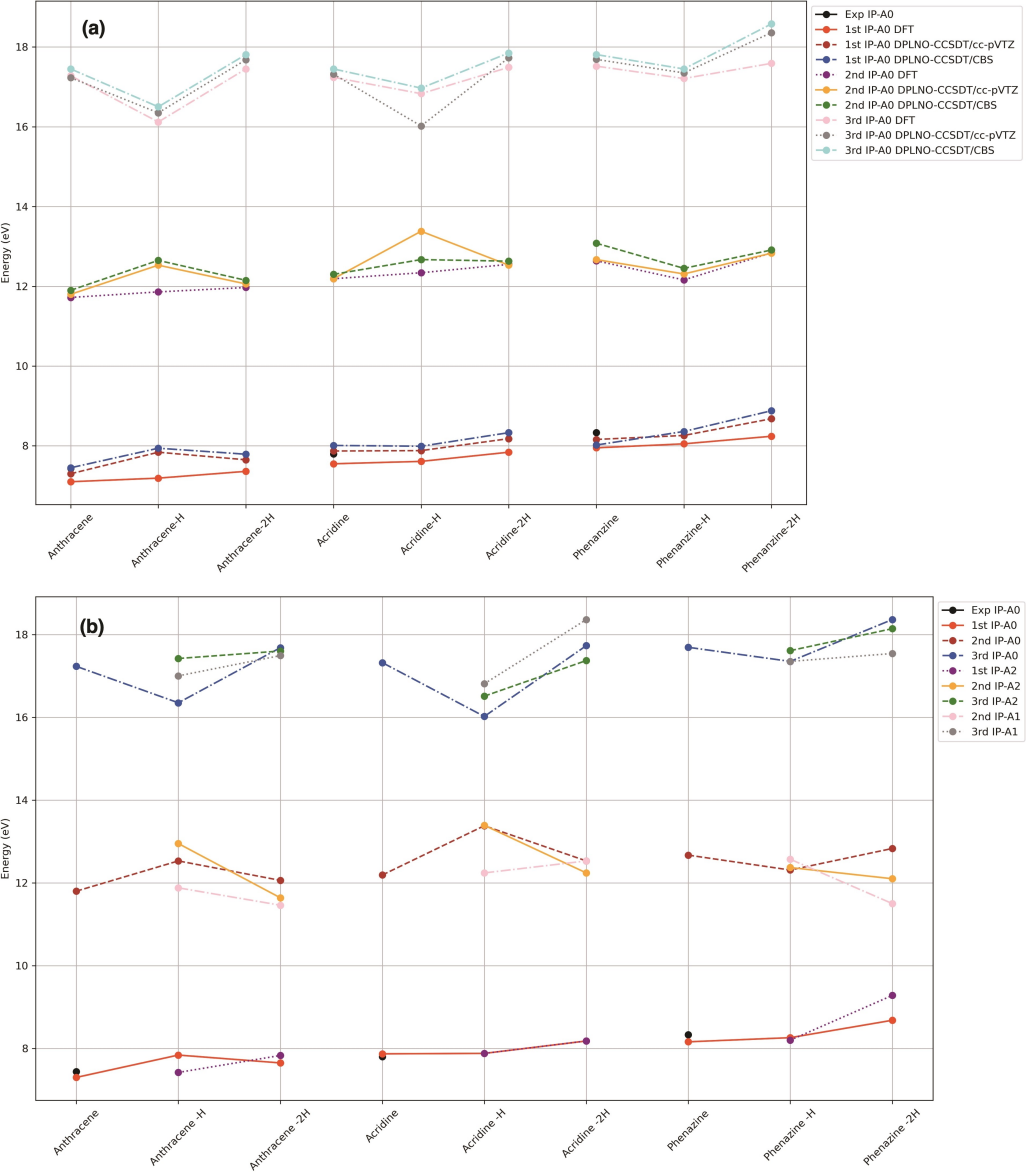
(a) The Ionization Potential IP−A0 estimated at different level of calculations of all the species under study. (b) Evolution of Ionization Potentials (IP−A0, IP−A1 and IP−A2) as a function of the degree of ionization at DLPNO‐CCSD(T)/cc‐pVTZ//B3LYP‐6‐311+G(d,p) level of theory. Experimental values were obtained from NIST.^[52]^

Before discussing the evolution of the IP across different compounds, we begin with a brief analysis of the differences observed between each calculation method (Figure 7a). For the first IP (1^st^ IP−A0), DFT exhibits a similar trend to the DLPNO‐CCSD(T) method using both the cc‐pVTZ basis and the CBS extrapolation, but in general B3LYP calculations underestimate 1^st^ IPs by 5–8 %. However, variations become more pronounced when estimating the second and third IPs, especially when assessing the loss of a single hydrogen atom. For instance, the difference in the 2^nd^ IP−A0 of acridine‐H between DFT and DLPNO‐CCSD(T)/CBS is approximately 0.4 eV, while the 3^rd^IP−A0 of the same compound shows only a minimal difference of 0.2 eV. In contrast, comparing DFT with DLPNO‐CCSD(T)/cc‐pVTZ yields a difference of about 0.8 eV for the 3^rd^ IP−A0. Overall, although DFT provides acceptable agreement with DLPNO‐CCSD(T) estimates when using either the cc‐pVTZ basis or the Complete Basis Set (CBS) extrapolation, relevant deviations are found for particular systems. When comparing experimental and theoretical results, DLPNO‐CCSD(T)/cc‐pVTZ aligns most closely with the experimental values and captures the observed trends. Consequently, our subsequent discussion will focus on the results obtained with this method. The evolution of the calculated IPs at this level is presented in Figure 7b.

A general observation of the results shows that the differences between the A0 and A2 IPs are practically negligible for the first IPs. For the second IPs, where the variation is larger, it remains arround 0.5 eV, indicating that electronic effects dominate over structural changes due to isomerization.

Focusing on the A0‐IP and observing the variation of the IPs with respect to the degree of dehydrogenation, the first IP tends to increase as hydrogens are lost. This increase is quite small (0.1 eV) for the first loss of hydrogen and around 0.2 eV for the second. For the second IP, there is not a clear trend, in anthracene and acridine increases for the loss of one H and decreases when two hydrogens are lost. phenazine‐H shows a significantly lower IP compared to phenazine and phenazine‐2H. The third IP always shows an oscillation, with the IP of the single‐dehydrogenated form being significantly lower.

Another interesting aspect is that the first IP significantly increases for all species (parent compound, ‐H, and −2H) with the presence of extra nitrogen atoms in the ring, with a systematic increase of approximately 0.5 eV per nitrogen.

### Dehydrogenation and Deprotonation Reactions

**Figure 8:**
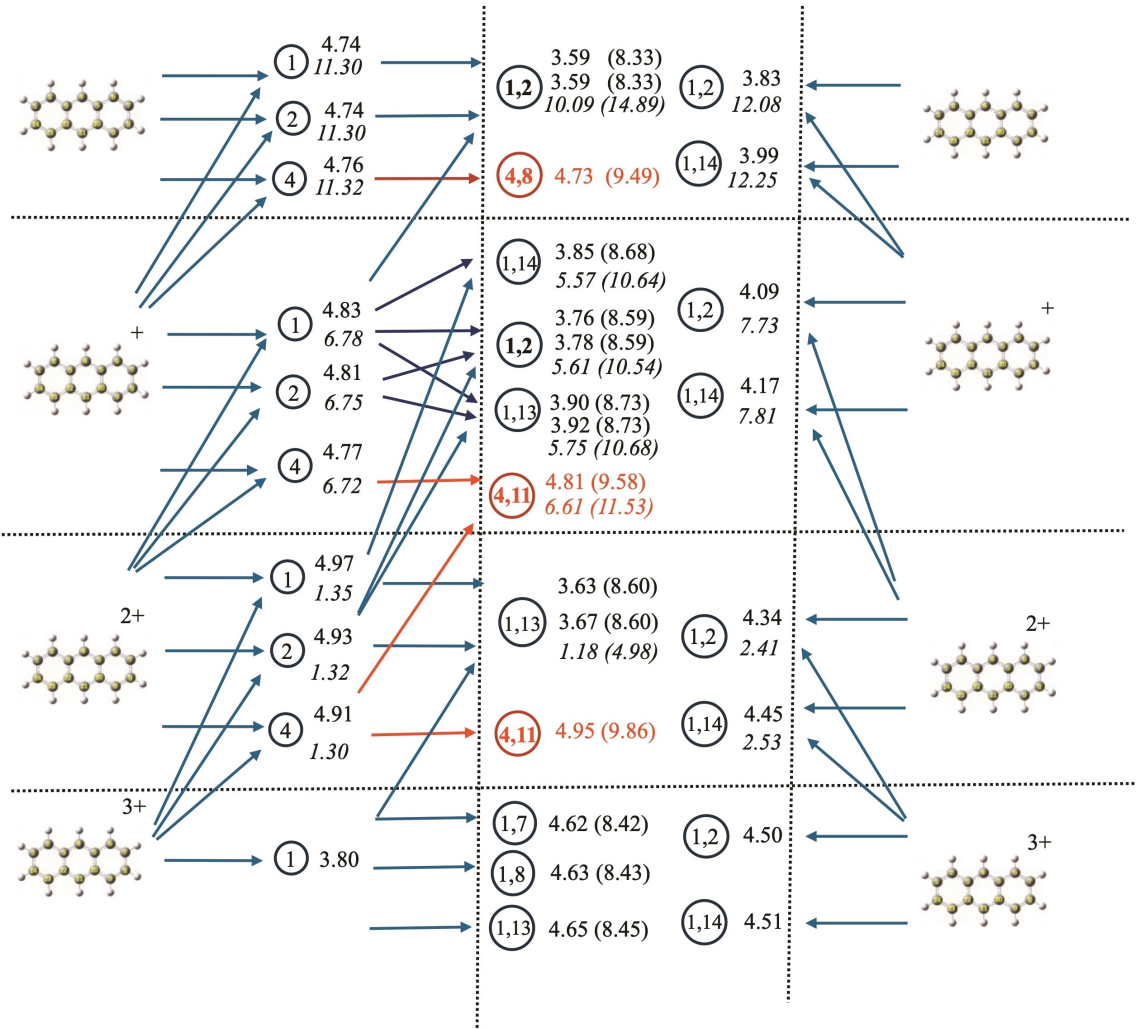
Reaction network for anthracene, reaction energies are given in eV. See text for explanation of the notation followed.

**Figure 9:**
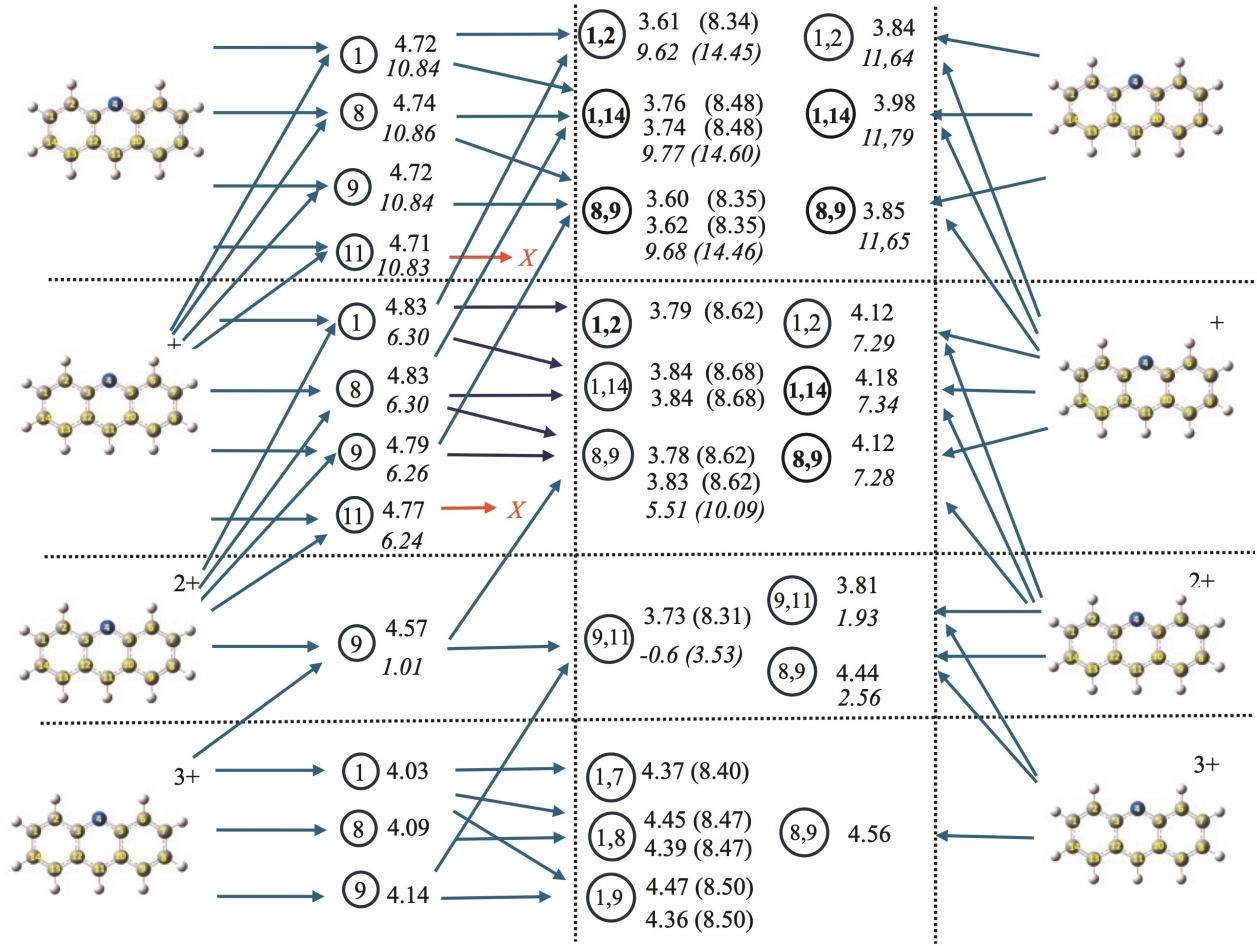
Reaction network for acridine, reaction energies are given in eV. See text for explanation of the notation followed

**Figure 10:**
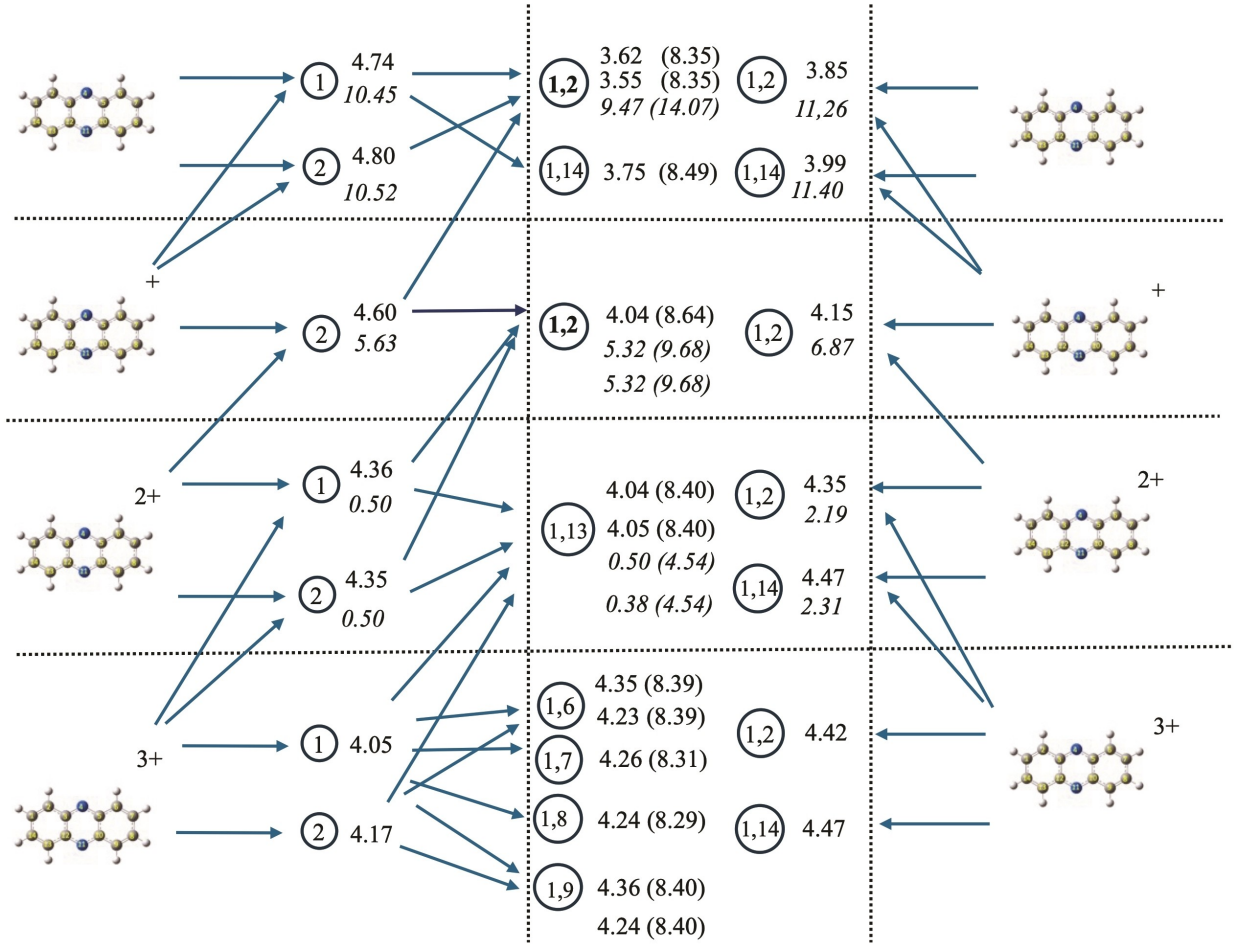
Reaction network for phenazine, reaction energies are given in eV. See text for explanation of the notation followed.

One of the most interesting outcomes of systematically studying all isomers of neutral and charged species is the ability to determine the different paths that can be followed in the hydrogenation process. The complex connections among different minima, usually referred to as the reaction network, are illustrated in Figures 8, 9, and 10 for anthracene, acridine, and phenazine, respectively. In these figures, the upper part represents the neutral species, while the lowest part represents the triply charged species. Species with different charges are separated by horizontal lines. Moving from left to right, the first column corresponds to the parent compounds, the second column to the singly dehydrogenated species (with the encircled number identifying the position of hydrogen loss). The central column corresponds to the doubly dehydrogenated species (with the encircled numbers indicating the positions from which the two hydrogens are lost). The horizontal arrows moving left to right represent reactions considering sequential loss of hydrogens (isomerization reactions are not considered in the figures). The last column, again representing the parent compound, includes horizontal arrows moving right to left that represent the simultaneous loss of two hydrogens to form H_2_. As discussed in the referenced study on coronene,^[82]^ direct loss of H_2_ can only occur if hydrogens are lost from adjacent carbon atoms and involves several steps with a total energy barrier of approximately 5 eV when the global charge is between 0 and 3. Loss from positions 2 and 4 is also possible but, in coronene, involves energy barriers 1 eV higher^[82]^ and has not been considered in the figures, as we expect the energy barriers for H_2_ loss to be similar for different PAHs. To simplify the schemes, only the most stable isomers for each charge have been considered, i. e., those within a range of approximately 10 kJ/mol (0.1 eV) according to Tables 1, 2, 3. The figures also visualize the processes corresponding to the loss of an H^+^/H_2_^+^, represented by ascending arrows connecting two different structures. The energies for all processes (in eV) are given to the right of the circle identifying the final product. Numbers in normal characters correspond to the loss of neutral H (horizontal arrows), and those in italics correspond to the loss of H^+^/H_2_^+^ (ascending arrows). When several numbers are given, they correspond to the energies of the different processes leading to this product (in the same order as the corresponding arrows arriving at it). In the central column, numbers in parentheses give the total energy for the sequential loss of two hydrogens. Finally, red arrows indicate dead ends, i. e., reactions that are possible but would involve an energy change of 1 eV higher than alternative routes and can be reasonably discarded.

Let's first consider the reaction network for anthracene (Figure 8). Starting from neutral anthracene (upper left corner in Figure 8), the loss of one hydrogen from positions 1, 2, or 4 are nearly degenerate processes, reflecting the degeneracy of anthracene‐H isomers discussed in Table 1. This implies that all hydrogens in anthracene should have nearly identical probabilities of being lost. However, a second hydrogen loss is only feasible from positions 1 or 2, leading to the same isomer (1,2). The alternatives (1,14) or (2,14), not represented in the figure, are 0.15 and 0.55 eV higher in energy, respectively, according to Table 1. From position 4, any option for a second hydrogen loss is more than 1.1 eV higher in energy. Dehydrogenation leading to isomer (4,8) is the most stable option from position 4, but it still represents 1.1 eV more energy than the (1,2) losses. This value is indicated in Figure 7 to emphasize that dehydrogenation from position 4 is a dead end. Therefore, even though it is expected that the first hydrogen in anthracene could be extracted from any position with practically equal probabilities, there is only one reasonable final isomer for the sequential loss of two hydrogens. These findings indicate that the loss of two adjacent hydrogens in anthracene has a significant stabilization effect when the molecule is neutral, compared to other alternatives. This is primarily because it helps maintain aromaticity in the remaining rings and allows the formation of a bond with partial triple character between both atoms.

For singly charged anthracene, the first hydrogen loss is slightly favored at position 4 (0.04–0.06 eV more stable than positions 2 and 1). However, this represents a dead end, since the second loss leading to the most stable isomer from position 4 (4,11) is much less favorable than second losses starting from positions 1 or 2. In these latter cases, the second loss leads to isomer (1,2) as the most favorable outcome. Two other possibilities close in energy lead to isomers (1,14) and (1,13). This indicates that losing two hydrogens from adjacent positions remains the preferable pathway, but non‐adjacent positions are becoming energetically competitive. The aromatic factor, keeping both losses within the same ring, still dominates. For doubly charged anthracene, the loss of the first hydrogen is quite degenerate across all positions. However, when two hydrogens are lost, a specific isomer is clearly favored. Unlike the previous cases, the preference now shifts to losing hydrogens from non‐adjacent carbons to minimize electronic repulsions, while still keeping them within the same ring to maintain aromaticity. This leads to (1,13) being the preferred isomer.

For the triply charged species, the situation is opposite. The first hydrogen loss is only favorable at position 1, as positions 2 and 4 are now significantly higher in energy (0.2 and 0.6 eV, respectively, see Table 1). For the second hydrogen loss, the criterion of maximizing charge separation prevails, with hydrogens being lost from opposite parts of the anthracene molecule. Although this reduces aromaticity, it significantly decreases charge repulsion. In this case, losses from positions (1,7) or (1,8) become nearly degenerate with (1,13). Any reaction leading to the loss of adjacent hydrogens, such as (1,2), is now very unstable due to maximized charge repulsions.

An interesting aspect reflected in the reaction network figures is the competition between the loss of a proton from the corresponding (q+1) species (ascending arrows: anthracene^(q+1)+^→anthracene^q+^ + H^+^) and the loss of H to yield the same compound (anthracene^q+^→anthracene^q+^ + H).

For the neutral the loss of H^+^ from anthracene^+^ represents a reaction path more than 2 times more energetic than the loss of H from neutral anthracene. In the case of the system singly charged, this path is nearly 2 eV above the loss of H and for doubly charged is the most efficient reaction path, of the order of 3.5 eV lower than the loss of H. Once again, the criteria of minimizing charge repulsion become dominant for charges greater than two.

All these findings are similar to those obtained previously for coronene,^[82]^ where the most stable structure for the loss of two hydrogens is from adjacent carbon atoms and with comparable energies (8.29, 9.14, and 8.70 eV for the neutral, singly charged, and doubly charged species, respectively). For the triply charged species, the loss of H^+^ is a more favorable channel. Finally, in the figure, we can compare the competition between the sequential loss of two hydrogens (the energy of the overall reaction is given in parentheses) and the direct loss of H_2_. The values for the latter reaction are always approximately 3.5 eV lower (3.83 vs 8.33 eV for the formation of 1,2‐dihydrogenated neutral anthracene). It is important to consider that this reaction forms a very stable H_2_ molecule instead of two H atoms. The trade‐off for the direct loss of H_2_ is that it involves a multistep process with a total energy barrier of approximately 5.00 eV. The evaluation of these mechanisms and energy barriers for anthracene, acridine, and phenazine is beyond the scope of this article. However, a comparison of the overall energy change for the loss of two hydrogens in coronene (3.51, 4.33, 3.91, 4.30 eV for q=0, 1, 2, 3) and anthracene (3.83, 4.09, 4.34, and 4.50 eV) suggests that similar barriers should be expected. In coronene, these barriers are 5.00, 5.04, 4.95, and 5.40 eV for q=0, 1, 2, 3, respectively.^[82]^ These values are much lower than the overall energy for sequential H loss, which is around 8.5 eV (see Figure 7). Therefore, the most efficient mechanism should be the loss of H_2_.

For acridine, the reaction network (Figure 9) becomes more complex due to the loss of symmetry, making many positions non‐equivalent. To avoid overloading the figure, we present only the most relevant positions. It is to say that the overall pattern remains quite similar to that of anthracene. For the loss of the first hydrogen, there are now five non‐equivalent hydrogens. The loss of any of them leads to reactions with very similar energies (approximately 4.7 eV), except for position 2 (not shown in the Figure), which has a slightly higher reaction energy (0.1 eV). Therefore, as in anthracene, it is expected that the first hydrogen can be lost with nearly equal probability from any position. For the loss of a second hydrogen, there are now three possibilities corresponding to the loss of adjacent hydrogens in one of the rings: (1,2), (1,14), or (8,9) (equivalent to (13,14)). The (1,2) and (8,9) pairs are nearly degenerate (both corresponding in anthracene to (1,2)), while (1,14) is 0.15 eV higher, similar to what was found in anthracene. Evolution from the central ring (position 11) represents a much higher energy route.

For singly charged acridine, the first hydrogen loss is slightly favored at the central position (now 11), but the difference with respect to any other position on the molecule is below 0.1 eV. As with anthracene, no further evolution is expected from position 11, as any possibility implies reaction energies higher than 0.8 eV compared to other channels. The three most stable channels for the second hydrogen loss are (1,2), (8,9), and (1,14), corresponding to adjacent positions, with (1,14) being slightly less favorable.

A notable deviation from anthracene is observed when acridine is doubly charged. In this state, the positions for the first hydrogen loss are no longer equivalent, with position 9 yielding a significantly more stable isomer than the others. This is likely due to the nitrogen in the central ring stabilizing the positive charge, with position 9 being the farthest away, thus favoring charge separation. From this point, only one reaction pathway dominates, corresponding to the loss of the second hydrogen from the central ring.

For the triply charged acridine, there are three possible reaction paths (from positions 1, 8, and 9) for the loss of the first hydrogen, all within an energy range of 0.1 eV. The second hydrogen is preferentially lost from a position further apart (positions 7, 8, or 9), all located in the opposite ring.

The loss of H^+^ becomes a competitive channel starting from q=2, and in general, reactions involving the loss of H^+^ have lower energies than their anthracene counterparts. Regarding the competition with H_2_ channels, the energies are quite similar to those of anthracene, suggesting they could be the most effective pathways. The energy barriers should be comparable to those of coronene, as the bonds involved in the reactions are all CH bonds, and the overall energy landscape is not very different.^[82]^ In the case of (acridine‐H_2_)^+2^, the most stable isomer (positions 9,11) could be reached by a direct H_2_ loss. The energy barriers for the equivalent position in doubly charged coronene have been studied and found to be 1 eV higher than those corresponding to the loss from adjacent carbons. Given that the energy difference between the (9,11) and (8,9) isomers is 0.63 eV, this route cannot be completely ruled out.

Finally, in phenazine, due to its higher symmetry and the absence of hydrogen in the central positions, the reaction network diagram is simpler (see Figure 10). For single hydrogen loss, position 1 is slightly favored in the neutral state. For singly charged phenazine, the loss from position 2 has much lower reaction energies. In the doubly charged state, both channels are degenerate, and for the triply charged state, position 1 is favored.

Regarding the loss of two hydrogens, similar factors to those described for anthracene and acridine explain the most favorable channels. For the neutral and singly charged states, the two hydrogens are lost from adjacent carbons, with (1,2) and (1,14) being the only possibilities, and (1,2) involving the lower energy. For the doubly charged state, both hydrogens are lost from the same ring but from non‐adjacent positions (1,13). All channels involving the loss from two different rings are competitive. As with anthracene and acridine, the loss of H^+^ becomes the preferential channel starting from q=2. The direct loss of H_2_ is most likely the channel with the lowest global energy, even leading, in the case of q=3, to isomers that are not the most stable ones.

## Conclusions

In this study, we systematically explored the stability and isomerism of neutral and dehydrogenated polycyclic aromatic hydrocarbons (PAHs) in various charge states, focusing on anthracene, acridine, and phenazine. Our findings highlight several key aspects that contribute to a deeper understanding of the reactivity and stability of these molecules, relevant in both laboratory and astrophysical contexts.

The structural symmetry and the presence of nitrogen atoms significantly impact the stability and reactivity of PAHs. In anthracene, the loss of hydrogens from adjacent positions is predominantly favorable, while in acridine and phenazine, the presence of nitrogen alters the most stable positions for dehydrogenation. Regarding the optimal site for the first dehydrogenation, there are various scenarios depending on the charge state. For the loss of a single hydrogen in the neutral state, nearly all positions in anthracene are degenerate. In acridine, all positions are nearly degenerate except for position 2, due to the proximity of the nitrogen atom. This also explains why, in phenazine, the loss of hydrogen from position 1 is more favorable than from position 2. For the cation, hydrogen loss from the central ring is more favorable, supported by resonance stabilization within the terminal rings of the singly charged species. For the dication, a significant electronic reorganization is observed, with anthracene and phenazine maintaining the same positions (4 and 2 respectively), while in acridine it shifts to position 9. For the trication, position 1 is always preferred for hydrogen loss.

In the case of losing two hydrogens, there is a clear competition between low and high spin states depending on the positions of the lost hydrogens. As a general rule, for the neutral and cation states, when both hydrogens are extracted from the same ring and from positions that differ by one or two C−C bonds, the low spin states are the most stable. In other cases, the high spin state is more stable, reflecting that the electrons are disconnected from each other. For the dication, the competition is tighter, and the two spin configurations are very close in energy, making it difficult to predict the most stable state. This is also observed in acridine and phenazine, where the presence of nitrogen atoms makes the loss and high spin state more degenerate. For the trication, the low spin state is always the most stable, regardless of the positions.

An important aspect for astrochemistry is the analysis of the infrared spectra of the compounds under study. The loss of hydrogen in anthracene shows characteristic frequencies of conjugated C_sp2_−C_sp2_ bonds. In acridine, the loss of hydrogen leads to three distinct structures depending on the charge, with differentiated vibrational frequencies. The spectral profiles of phenazine‐H resemble those of anthracene‐H in the neutral state. For the elimination of two hydrogens, various structures are observed in different molecules. In anthracene, acridine, and phenazine with zero and one charge, the elimination of H_2_ occurs at adjacent carbons, creating a C−C bond similar to a triple bond with a corresponding vibration frequency. For the triply charged structures, the loss of two hydrogens occurs at symmetrical positions within the molecule, making the tricationic forms without two hydrogens resemble their trication‐H forms, with similarities in their vibrational spectra in the range of 1700–1780 cm^−1^.

We also systematically explored the stability and reaction pathways of these compounds in various charge states. Our findings highlight the significant impact of structural symmetry and the presence of nitrogen atoms on the stability and reactivity of these molecules. The reaction networks constructed for anthracene, acridine, and phenazine reveal preferred pathways for hydrogen loss, influenced by the need to minimize charge repulsion and maintain aromaticity.

Key insights include the predominance of adjacent hydrogen loss in neutral and singly charged states, and the shift towards non‐adjacent hydrogen loss in doubly and triply charged states to reduce electronic repulsion. Additionally, the competition between sequential hydrogen loss and direct H_2_ loss indicates that the latter is generally the more energetically favorable pathway, especially in highly charged states.

Our research also underscores the potential relevance of these findings in astrophysical environments, where high‐energy collisions could produce unique PAH structures not typically observed in laboratory settings. Studying spin states and reaction intermediaries in these exotic conditions provides valuable information for astrochemists seeking to understand the complex chemistry of the interstellar medium.

By expanding our understanding of PAH reactivity and stability under various conditions, this work contributes to the broader field of astrochemistry and offers new directions for future research in both laboratory and extraterrestrial contexts.

## Conflict of Interests

There are no conflicts to declare.

1

## Data Availability

The Data for this article are available at https://doi.org/10.19061/iochem‐bd‐6‐387.
